# A Novel Role of Connective Tissue Growth Factor in the Regulation of the Epithelial Phenotype

**DOI:** 10.3390/cancers15194834

**Published:** 2023-10-02

**Authors:** Radhika P. Gogoi, Sandra Galoforo, Alexandra Fox, Colton Morris, Harry Ramos, Vir K. Gogoi, Hussein Chehade, Nicholas K. Adzibolosu, Chenjun Shi, Jitao Zhang, Roslyn Tedja, Robert Morris, Ayesha B. Alvero, Gil Mor

**Affiliations:** 1 Karmanos Cancer Institute, Wayne State University, 4100 John R St, Detroit, MI 48202, USA; rmorris@med.wayne.edu; 2 C.S. Mott Center for Human Growth and Development, Department of Obstetrics and Gynecology, Wayne State University, Detroit, MI 48202, USA; sandra.galoforo@wayne.edu (S.G.); alexandra.fox@wayne.edu (A.F.); cjmorris20@gmail.com (C.M.); harryramos@wayne.edu (H.R.); gogoi.vir@gmail.com (V.K.G.); gm0971@wayne.edu (H.C.); hc7424@wayne.edu (N.K.A.); roslyntedja@wayne.edu (R.T.); ayesha.alvero@wayne.edu (A.B.A.); 3 Department of Biomedical Engineering, Wayne State University, Detroit, MI 48202, USA; chenjun.shi@wayne.edu (C.S.); zhang4@wayne.edu (J.Z.)

**Keywords:** epithelial–mesenchymal transition, cytoskeleton, extracellular matrix remodeling, ovarian cancer, anoikis resistance, metastasis

## Abstract

**Simple Summary:**

Cancer progression and metastasis is associated with the transformation of epithelial cells into mesenchymal cells, a process known as epithelial–mesenchymal transition (EMT). We have a better understanding of the genes promoting EMT; unfortunately, our understanding of the genes responsible for maintaining the epithelial phenotype is limited. Our objective was to better understand the mechanisms preventing epithelial cells from undergoing EMT. We identified CTGF as an essential gene controlling the epithelial phenotype. Its loss is associated with the early cellular modifications required for EMT.

**Abstract:**

Background: Epithelial–mesenchymal transition (EMT) is a biological process where epithelial cells lose their adhesive properties and gain invasive, metastatic, and mesenchymal properties. Maintaining the balance between the epithelial and mesenchymal stage is essential for tissue homeostasis. Many of the genes promoting mesenchymal transformation have been identified; however, our understanding of the genes responsible for maintaining the epithelial phenotype is limited. Our objective was to identify the genes responsible for maintaining the epithelial phenotype and inhibiting EMT. Methods: RNA seq was performed using an vitro model of EMT. CTGF expression was determined via qPCR and Western blot analysis. The knockout of CTGF was completed using the CTGF sgRNA CRISPR/CAS9. The tumorigenic potential was determined using NCG mice. Results: The knockout of CTGF in epithelial ovarian cancer cells leads to the acquisition of functional characteristics associated with the mesenchymal phenotype such as anoikis resistance, cytoskeleton remodeling, increased cell stiffness, and the acquisition of invasion and tumorigenic capacity. Conclusions: We identified CTGF is an important regulator of the epithelial phenotype, and its loss is associated with the early cellular modifications required for EMT. We describe a novel role for CTGF, regulating cytoskeleton and the extracellular matrix interactions necessary for the conservation of epithelial structure and function. These findings provide a new window into understanding the early stages of mesenchymal transformation.

## 1. Introduction

Epithelial–mesenchymal transition (EMT) is a biological process where epithelial cells lose their adhesive properties and gain invasive, metastatic, and mesenchymal properties [1]. EMT plays an important role during embryo development and has been characterized in tumor formation and the metastatic process. Growing evidence suggests that EMT is not a simple one-directional process wherein epithelial (E) cells become mesenchymal (M) cells, but is a dynamic process that involves multiple stages of differentiation and adaptation. In addition, many of these intermediate stages reveal a high degree of plasticity. 

EMT involves multiple steps which start with: (1) the loss of apical–basal cell polarity; (2) the disruption of cell-to-cell interaction; (3) extracellular matrix (ECM) disassembly resulting in the degradation of the basal membrane; and (4) ECM reorganization, followed by (5) the reorganization of the actin cytoskeleton [1]. Several transcription factors including genes in the SNAIL, TWIST, and Zeb families have been described as the hallmarks of mesenchymal transformation [2]; however, they may not play a role in the early stages of cytoskeletal remodeling, the loss of apical–basal polarity, cell–cell adhesion weakening, and the loss of integrity of the basement membrane. The characteristics of these early EMT cells, their importance in tumor progression, and the key regulators in the tumor microenvironment that support this phenotype are still poorly understood. 

Ovarian cancer (OC) is the fifth leading cause of cancer deaths among women in the United States, with approximately 22,000 women diagnosed and 14,000 women dying from the disease annually. Four out of five women with OC are diagnosed in the advanced stages with a precipitous drop in 5-year survival from 95% to between 35 and 60% in advanced-stage disease [3]. Unfortunately, the biological components of the early stages of malignant transformation and metastatic process in ovarian cancer have not been elucidated. 

The origin of the High-Grade Serous Ovarian Cancers (HGSOC) is widely accepted to be the fallopian tubes, where early lesions have been identified and are known as serous tubal intraepithelial carcinoma (STIC) or serous tubal intraepithelial lesions (STILs). The main characteristic of these lesions is the presence of a “p53” signature determined through immunocytochemistry [4,5]. It is thought that the early stages of EMT occur at the STIC or STILs, generating mesenchymal cells that migrate into the ovaries during ovulation [6] and the peritoneal cavity [7]. These findings suggest that the early events associated with EMT may occur within the fallopian tube epithelium [4]. Thus, the identification of this “trigger/s”, its downstream targets, and therapeutic targeting are essential in efforts to substantively improve the survival of women with OC. 

Connective tissue growth factor (CTGF) is a secreted extracellular protein encoded by the *CCN2* gene. It belongs to the CCN family of extracellular-matrix-associated heparin binding proteins and has been implicated in the control of a number of biological processes including cell proliferation, differentiation, and adhesion [8,9]. Secreted CTGF can bind to several cell surface receptors including integrins, heparin sulfate proteoglycans, lipoprotein-receptor-related proteins, and tyrosine kinase receptors functioning as a bridge between the ECM and the epithelial structure of the cells [10]. The interaction between the epithelial cell and the extra-cellular matrix components including laminin, collagen, and integrins has been shown to be important in regulating EMT [11,12]. 

Using an in vitro model of ovarian cancer EMT, we identified CTGF as one of the genes differentially expressed during the early stages of epithelial differentiation towards a mesenchymal phenotype. We hypothesized that CTGF, by maintaining the interaction between the epithelium and the ECM, preserves their epithelial phenotype. The overall objective of this study was to better understand the molecular changes associated with the early transition between the epithelial and mesenchymal phenotype in OC and, more specifically, to elucidate the role of CTGF in this transition. Using in vitro and in vivo studies, we demonstrate that the loss of CTGF expression is associated with cytoskeleton reorganization, ECM remodeling, and the acquirement of early mesenchymal properties.

## 2. Materials and Methods

Cell lines and culture conditions: Samples were obtained with patient consent and approved by the Human Investigation committee of the Yale University School of Medicine. Ovarian cancer cells were isolated from patients with stage III or IV high-grade serous carcinoma. These in-house-derived ovarian cancer cells (R182, R2615, MR182, and MR2615) have been previously described [13,14,15,16]. Ovarian cancer cell lines (OVCAR3, OVCAR432, OVCAR433, SKOV3, and A2780) were purchased from ATCC (Manassas, Virginia). Cell lines were cultured in RPMI1640 (ThermoFisher Scientific, Waltham, MA, USA), supplemented with 10% FBS, and 1% each of Na pyruvate, HEPES, MEM-NEAA, and penicillin-streptomycin under standard cell culture conditions. All cell lines were tested regularly for mycoplasma via PCR and authenticated once a year via STR profiling and used within 6 passages between experiments.

Antibodies and reagents: The antibodies used are listed in Supplementary Table S1. Phalloidin iFluor488 (Abcam, Boston, MA, USA) was used for f-actin staining. Recombinant human CTGF (rCTGF) was purchased from PeproTech (Cranbury, NJ, USA). 

Anoikis Assay: The anoikis assay was performed in a 6- or 96-well Costar cell culture plate with Ultra-low attachment surface (Corning, Kennebunk, ME, USA). To determine cell viability, in the 96-well plate, 10,000 cells were plated/well in 200 μL growth media. At 0, 24, 48, and 72 h of culture, 40 μL of Cell Titer 96 AqueousOne Solution (Promega, Madison, WI, USA) was added to triplicate wells (including wells with media only), incubated for 4 h @ 37 °C, and then absorbance readings were taken at 450 nm. Data are presented as relative cell viability (1 = cell viability at time = 0). 

Conditioned media protocol: R182 and R182 CTGFKO cells were plated into 6-well TC plates at 4 × 10^5^/well in growth media. When cells were completely confluent (24 h), media were exchanged for Optimem media (Gibco, Billings, MT, USA), 4 mL/well. After 24 h, media were collected from cells (=conditioned media), centrifuged at 1500 rpm, 10 min, 4 °C, and supernatant was transferred to a new tube. Conditioned media were either frozen immediately at −80 °C or concentrated. Conditioned media were concentrated using Amicon Ultra4-Centrifugal Filter (Millipore, Billerica, MA, USA), 30 kD cutoff. An amount of 4 mL of conditioned media was added to the filter and centrifuged at 4000× *g*, 15 min at RT. Concentrate was collected and volume was measured. Concentrating resulted in a 60–88-fold concentrate.

CRISPR: Knockout of CTGF was completed using the CTGF sgRNA CRISPR/CAS9 All-in-One Lentivector set (Human) (Applied Biological Materials Inc., Richmond, BC, Canada), which includes 3 different guides. Selection for knockout was with puromycin in a mixed population of cells. CTGF knockout was confirmed with Sanger sequencing. Genomic DNA was extracted from the CTGF k-o cells with Qiagen DNAeasy Blood & Tissue kit. A PCR with CTGF primers was completed and the pcr product sent to Genewiz LLC (South Plainfield, NJ, USA) for sequencing.

IC50 assay chemosensitivity. IC50 was calculated as described previously [17].

Invasion assay: Invasion assays were performed in 96-well plates. Briefly, 3000 cells were plated in 50% Cultrex-BME, RGF (R&D Systems, Inc., Minneapolis, MN, USA)/RPMI1640 cell culture media/well of a 96-well plate and then placed in a humidified (37 °C, 5% CO_2_) BioTek BioSpa live cell analysis system (BioTek, Santa Clara, CA, USA). Cells were monitored and imaged every 4 h for a total of 6 days using a Cytation 5 cell imaging system (BioTek, Santa Clara, CA, USA) and confluence was determined.

RNA sequencing: RNA sequencing of CTGF knockout (KO) and wild-type (WT) R182 cells were performed by GENEWIZ LLC (South Plainfield, NJ, USA) as described previously [18]. Differential gene expression analysis comparing R182-CTGFKO cells to WT R182-WT cells was performed by GENEWIZ LLC using the DESeq2 package [19]. Briefly, a generalized linear model based on the two groups being compared was fitted to the gene expression data with Wald’s test performed to compute fold changes and corresponding p-values for each gene [19]. Genes were considered differentially expressed if the corresponding FDR-adjusted *p*-value was <0.05 and absolute log2 fold change >0.6. Gene Ontology analysis was performed using the iPathwayGuide [20,21,22] software from AdvaitaBio (Advaita Corporation, Ann Arbor, MI, USA). Gene Ontology biological process terms were considered significantly enriched if the respective FDR-adjusted *p*-values were <0.05. The enriched biological processes as well as the volcano plot of differentially expressed genes were graphed using the ggplot2 package in R [23].

q-PCR: Total RNA was extracted using the RNeasy Mini Kits (Qiagen, Austin, TX, USA) according to manufacturer’s instructions. cDNA was synthesized using iScript cDNA kit (Bio-Rad, Hercules, CA, USA). Quantitative PCR was performed followed by detection with the CFX96TM PCR detection system (Bio-Rad, Hercules, CA, USA). GAPDH was used as the reference control. Relative expression was calculated using the comparative ΔΔCT method. All experiments were repeated in triplicate. Primer sequences are included in the Supplementary Materials.

Protein extraction and quantification: Protein extraction and quantification was completed using BCA assay as previously described [24].

SDS polyacrylamide gel electrophoresis and Western blot analysis: Protein samples were denatured in sample buffer and underwent electrophoresis on a 12% SDS-polyacrylamide gel as previously described. The blots were imaged using GE ImageQuant LAS 500 chemiluminescence (Cytiva Life Sciences, Marlborough, MA, USA).

Immunofluorescence and Microscopy: For immunofluorescence, cells were grown and treated in 4-chamber cell culture slides (Lab-Tek, ThermoFisher, Waltham, MA, USA). Cells were fixed with 4% paraformaldehyde and permeabilized with 0.3% TritonX-100/PBS. After washing with PBS, cells were incubated overnight @ 4 °C with 1° antibody (in 3%BSA/PBS), washed with PBS, then incubated with 2° antibody (in 3%BSA/PBS), 1 h at room temperature. After washing with PBS, chambers were removed from slide and coverslip was mounted with SlowFade Diamond Antifade Mountant with DAPI (Invitrogen, Eugene, OR, USA). Slides were then imaged on a fluorescent microscope (Nikon Eclipse 90i, Nikon, Melville, NY, USA) with pictures taken (NIS Elements AR, Nikon, Melville, NY, USA).

Stiffness Assay: The mechanical properties of the cells were quantified using a confocal Brillouin microscope. The detail of the instrumentation has been reported before [25]. In brief, the instrument consists of a laser source (660 nm, Laser Quantum, Stockport, UK), a standard microscope (IX83, Olympus, Tokyo, Japan), a home-built Brillouin spectrometer, and optical components that were used to guide the laser beam into the microscope and collect backward-scattered Brillouin signals. The measured Brillouin frequency shift $\omega_{B}$ is related to the elastic longitudinal modulus $M^{\prime }$ with the equation $\omega_{B}=2n/\lambda \cdot \sqrt{M\prime /\rho }$, where λ is laser wavelength and n and ρ are refractive index and density of the material. For cells in different physiological conditions, the ratio $n/\sqrt{\rho }$ can be approximated as constant. Therefore, we here can use Brillouin shift to estimate the cellular mechanical properties. 

Tumorigenic potential in vivo: All of the in vivo studies described herein were approved by Wayne State University Animal Care and Use Committee. To assess tumorigenic potential, 5 million or 20 million R182 WT or R182 CTGF KO cells were injected into NCG mice (Charles River, NCG strain 572, NOD-Prkdc^em26Cd52^Il2rg^em26Cd22^/NjuCrl) in a 50:50 mixture of Hydrogel (TheWell Bioscience TWG001, North Brunswick, NJ, USA) and cell suspension in 1× PBS. Cells in Hydrogel were injected into the right flank of each mouse in a volume of 100 μL. Subcutaneous volume was assessed every 2–3 days by caliper and subcutaneous volume was calculated as {(W × (H^2^)}/2 mm^3^. Animals were sacrificed once a time point of 46 days was reached. 

Statistical Analysis: The unpaired two-tailed *t*-test was used for comparison between different groups. Data from each group were collected with at least 3 biological repeats. *p* values of less than 0.05 were considered statistically significant. All statistical analyses were performed using Prism 9 software (GraphPad Software, San Diego, CA, USA). Illustrations were created using Biorender^TM^ software.

## 3. Results

### 3.1. Differential Expression of CTGF during Early EMT

**Figure 1:**
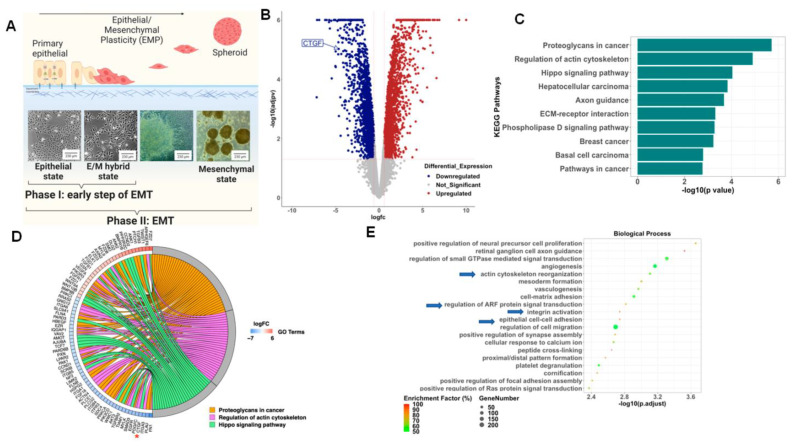
Differentially expressed pathways and biological processes in epithelial ovarian cancer cells and cells in the E/M hybrid state. (**A**). Model outlining the process of epithelial–mesenchymal plasticity in R182 ovarian cancer cells as described previously in our lab by Tedja et al. [15]. (**B**). Volcano plot of differentially expressed genes (DEGs). Blue dots represent downregulated DEGs and red dots represent upregulated DEGs. (**C**). Bar plot of top 10 differentially regulated pathways. (**D**). Chord diagram of top three differentially regulated pathways and their associated DEGs (Star points to CTGF). (**E**). Top 20 differentially regulated biological processes (arrows highlight processes associated with ECM reorganization).

To better understand the early modifications associated with the process of EMT, we analyzed the transcriptome of epithelial ovarian cancer cells (EOCCs) undergoing mesenchymal transformation using a previously reported in vitro model of EMT [15]. In short, the EOCCs (clones R182 and R2615, anoikis sensitive, non-invasive, and non-tumorigenic) cultured in high confluence in low serum conditions for 12 days undergo morphological changes characteristic of EMT [15]. In this model, we can define three stages of differentiation based on cellular morphology: (1) the epithelial state; (2) the E/M hybrid state (at 48 h); and (3) the spheroid or mesenchymal state (9–12 days) (Figure 1A). To characterize the molecular signature of cells in the E/M hybrid state, we performed bulk RNA sequencing and compared epithelial R182 EOCCs (CK18+/β-catenin+/Slug-/Twist1-) [16] with their corresponding E/M hybrid state of differentiation [15,17,26]. RNA sequencing data were analyzed utilizing the iPathwayGuide^TM^ software. We identified a total of 3860 DEGs (Figure 1B) at an absolute log2-fold change threshold of 0.6 and FDR-adjusted *p* value threshold of 0.05. The top 10 differentially regulated pathways are shown in Figure 1C,D, which shows a Chord diagram with DEGs for the top three differentially regulated pathways (proteoglycans in cancer, the regulation of actin cytoskeleton, and the HIPPO signaling pathway). Further analysis of the differentially regulated biological processes during this early stage of differentiation revealed processes associated with the remodeling of cytoskeleton, integrin activation, ARF signal transduction, and cell–cell adhesion (Figure 1E). Interestingly, the top KEGG pathways and GO biological process terms differentially impacted in the early stages of EMT are the regulation of the actin cytoskeleton and the HIPPO signaling pathway. Taken together, these data suggest that a characteristic of the early stage of EMT is associated with the regulation of the cytoskeleton and the interaction with the surrounding ECM. 

To better understand the gene(s) responsible for these changes, we analyzed the DEGs within the HIPPO pathway during early EMT and identified CTGF as significantly downregulated in the early mesenchymal stage compared to the epithelial stage (4-fold) (Figure 1D, asterisk). Given that CTGF is a validated downstream target of the HIPPO pathway and is known to play a role in cell differentiation [27], we focused on the validation and characterization of the role of CTGF during early EMT. 

**Figure 2:**
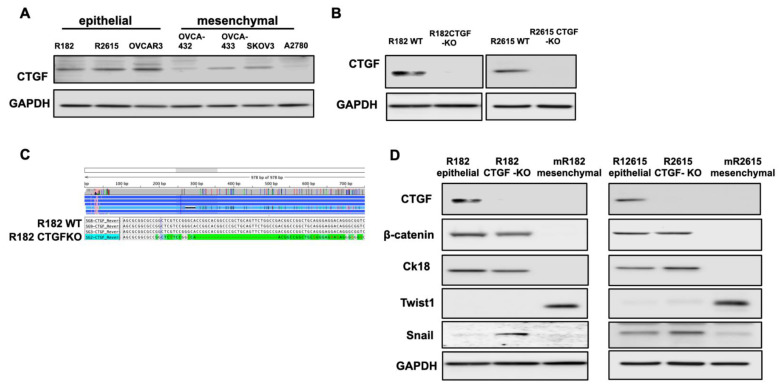
CTGF regulates epithelial and mesenchymal markers in ovarian cancer cells. (**A**). Western blot demonstrating CTGF expression in various OC cell lines. (**B**). Western blot demonstrating loss of CTGF by CRISPR KO in R182 OC cell lines. (**C**). Sanger sequencing verifying deletion of CTGF in R182 CTGF KO cell line. (**D**). Western blot evaluating expression of epithelial and mesenchymal markers in wild-type and CTGF-KO R182 cell lines. Representative figures of three independent experiments. N = 3. The uncropped blots are shown in Supplementary File S1.

To validate the findings from the RNA seq, we screened the panel of OCCs at different stages of differentiation. Clones R182, R2615, and OVCAR3 have epithelial characteristics [28,29,30], while OVCAR432, OVCA433, SKOv3, and A2780 have mesenchymal characteristics [28,29]. As shown in Figure 2A, CTGF is expressed in OCCs with epithelial characteristics and downregulated or not detectable in OCCs with mesenchymal phenotype as characterized by the expression of the epithelial markers B-catenin and CK18 and the expression of the early mesenchymal marker Snail, but not TWIST (Figure 2A). 

### 3.2. Loss of CTGF Reprograms ECM in Ovarian Cancer Cells

To better understand CTGF’s function, we knocked out CTGF in epithelial OCC clones R182 and R2615 using the CRISPR/Cas9 system. Figure 2B shows the successful KO of CTGF in the two cell lines using Western blot, and Figure 2C verifies the KO using Sanger sequencing.

Our next objective was to determine the biological changes associated with CTGF deletion in the EOCCs. We first characterized the expression of known epithelial and mesenchymal markers in the wild-type and CTGF-KO cell lines. CTGF-KO cells maintained the epithelial markers β-catenin and CK18, gained (R182 CTGF-KO) or upregulated (R2615 CTGF-KO) the mesenchymal marker Snail, and remained Twist1- (Figure 2D). The gain in Snail expression in CTGF-KO cells is interesting given that Snail is associated with the early stages of EMT [31,32]. This suggests that the lack of CTGF may be related to a transitional stage between the epithelial and mesenchymal phenotype. 

**Figure 3:**
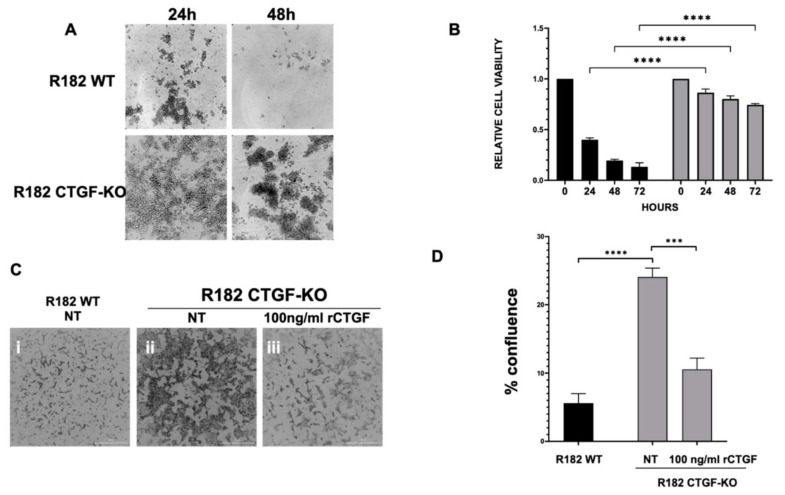
CTGF negatively regulates anoikis resistance. R182 WT and R182 CTG-KO cells were cultured in ultra-low attachment conditions. (**A**) Culture morphology was assessed via microscopy after 24 and 48 h. Scale bar 1000 μm. (**B**) Cell viability was quantified at designated time points using Celltiter96 assay. Experiments were performed independently and in triplicate. Data are presented as mean ± SEM and an unpaired *t*-test was used to calculate statistical significance. (**C**) R182 WT, R182 CTG-KO, and R182 CTG-KO cells treated with 100 ng/mL recombinant CTGF were cultured in 50% Matrigel. Culture morphology was assessed via microscopy at day 6. Scale bar 1000 μm. (**D**) Quantitation of invasion assay at 160 h. Independent experiments were performed in triplicate. Data are presented as mean ± SEM and an unpaired *t*-test was used to calculate statistical significance. *** *p* ≤ 0.001. **** *p* ≤ 0.0001.

One of the early adaptation changes observed during EMT is the acquisition of anoikis resistance. Anoikis resistance or the ability of a cell to resist apoptosis and survive in unattached conditions is an essential property of mesenchymal cells [33]. We hypothesized that the loss of CTGF expression may affect anoikis resistance. To test this hypothesis, clones of R182/R2615 CTGF-KO cells and R182/R2615 WT cells were grown in ultra-low attachment plates. While WT epithelial cells underwent cell death when cultured in low attachment plates, the loss of CTGF permitted OC cells to survive in detached conditions for up to 72 h (*p* < 0.01) (Figure 3A,B). Taken together, our findings so far demonstrate that CTGF is necessary to maintain an epithelial phenotype and that its loss confers anoikis resistance. 

Another phenotype acquired during EMT is the ability to invade. To determine if the loss of CTGF can confer invasion capacity, R182WT and R182 CTGF-KO cells were seeded in 50% Matrigel and invasion was monitored via live imaging and quantified by measuring cell culture confluence. We observed a significant increase in invasion capacity in R182 CTG-KO cells compared to R182 WT EOCCs by day 6 (*p* = 0.02; Figure 3C,D). Interestingly, this was abrogated with the addition of recombinant CTGF (Figure 3C,D), further proving that CTGF negatively regulates invasion. 

Next, we evaluated whether the lack of CTGF could affect the response of cancer cells to chemotherapy; however, we did not find any significant difference between the WT and CTGF-KO cells in their response to Cisplatin. Taken together, our data demonstrate that the loss of CTGF in OC cells leads to the acquisition of anoikis resistance and increased invasiveness, but did not impact their response to chemotherapy, which are properties supportive of the early changes associated with epithelial mesenchymal plasticity. 

### 3.3. Inhibition of CTGF Expression Is Associated with Changes on the Transcriptome of OCCs

**Figure 4:**
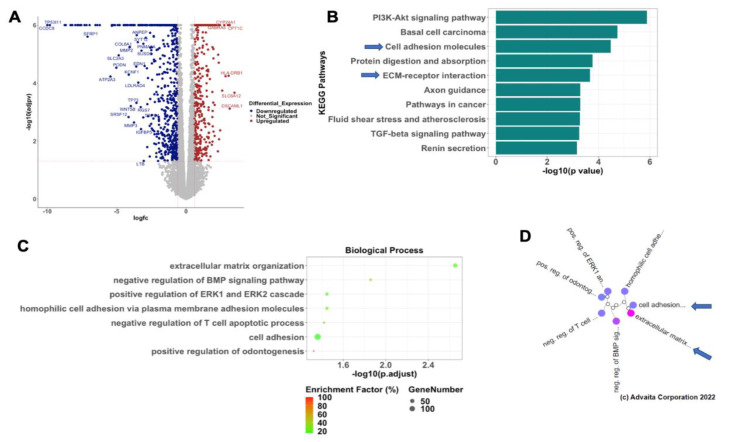
Loss of CTGF reprograms cell adhesion and ECM in OC cells. RNA sequencing was performed in R182 WT and R182 CTG_KO cells. (**A**) Volcano plot of differentially expressed genes (DEGs). Blue dots represent downregulated DEGs and red dots represent upregulated DEGs. (**B**) Bar plot of top 10 differentially regulated pathways. (**C**) Top seven differentially regulated biological processes. (**D**) Dendogram of top seven differentially regulated biological processes.

To better understand the molecular mechanism associated with the CTGF regulation of the epithelial phenotype, we performed RNA sequencing in WT and R182 CTGF-KO OC cells and compared their transcriptome. We found that the loss of CTGF in EOCCs is associated with significant changes on their transcriptome. Out of 14,054 genes with measured expression, we observed 1106 differentially expressed genes (DEGs; *p* < 0.05 and absolute log2-fold change >0.6) in the CTGF-KO OC cells. Of these DEGs, 385 were upregulated and 721 were downregulated (Figure 4A). Pathway Enrichment Analysis showed 47 significantly impacted pathways, with the PI3K-Akt pathway, basal cell carcinoma, cell adhesion molecules, protein digestion and absorption, and ECM–receptor interaction being the top five differentially regulated pathways (Figure 4B). Gene Ontology analysis identified the extracellular matrix organization as the most significantly regulated biological process associated with loss of CTGF (Figure 4C). Gene Ontology analysis with high specificity pruning identified the extracellular matrix organization, negative regulation of BMP signaling pathway, positive regulation of ERK1 and ERK2 cascade, homophilic cell adhesion via plasma membrane adhesion molecules, and negative regulation of T cell apoptotic process as the top five differentially regulated biological processes (Figure 4D). These findings further support the initial observation that early EMT involves cytoskeleton and ECM remodeling, and that CTGF is a central regulator of these changes. 

### 3.4. Loss of CTGF Alters Extracellular Matrix 

**Figure 5:**
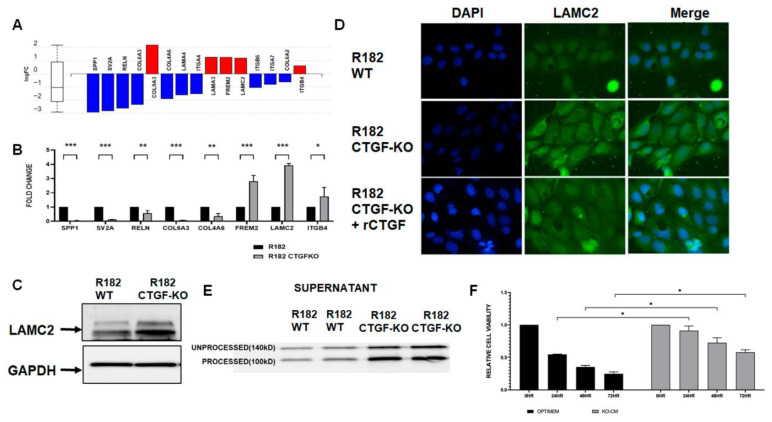
CTGF reprograms ECM–receptor interaction. (**A**) DEGs in the ECM–receptor interaction pathway comparing R81 WT and R182 CTGF-KO cells. (**B**) Validation of identified DEGs via qPCR. Data are presented as mean + SEM and an unpaired *t*-test was used to calculate statistical significance. *** *p* ≤ 0.001; ** *p* ≤ 0.01 and * *p* ≤ 0.05. (**C**) LAMC2 protein expression in cell lysate of WT and CTGF KO cells (arrow). (**D**) LAMC2 IF in R182 WT, R182 CTGF-KO, and R182CTGF-KO treated with 100 ng/mL rCTGF. Scale bar 10 μm. (**E**) Secreted LAMC2 protein expression in media of R182 WT and R182 CTGF-KO cells. (**F**) Addition of conditioned media from R182-CTGF KO cells to R182 WT cells confers anoikis resistance in R182 WT cells. Anoikis resistance was measured as described in the Materials and Methods section. Briefly, R182 cells were plated in either optimum or CTGF KO media and anoikis resistance was measured up to 72 h. Three independent experiments were performed in triplicate. Data are presented as mean ± SEM and an unpaired *t*-test was used to calculate statistical significance. * denotes *p* ≤ 0.01. The uncropped blots are shown in Supplementary File S1.

Given the significant representation of extracellular matrix reorganization in the CTGF KO cells (Figure 5A), we validated several DEGs in the ECM receptor interaction pathway via qPCR (Figure 5B). Similar to the findings in the RNA seq, the mRNA expression for SPP1, SV2A, RELN, COL6A3, and COL4A6 was significantly downregulated in the CTGF KO cells, while FREM2, LAMC2, and ITGB4 were upregulated in the CTGF KO cells (Figure 5B). Interestingly, mRNA expression levels for LAMC2, a gene encoding the gamma subunit of the Laminin332 of extracellular matrix proteins, was significantly upregulated (4X) in cells lacking CTGF (Figure 5B). To confirm that the changes in the mRNA are translated into protein, we evaluated LAMC2 protein expression via Western blot analysis and observed the increased expression of LAMC2 in CTGF KO cells compared to the WT (Figure 5C). Similarly, the immunofluorescence (IF) for LAMC2 showed enhanced staining in R182 CTGF-KO compared to R182 WT cells (Figure 5D). To confirm that the differential expression in LAMC2 was due to the absence of CTGF, we treated CTGF-KO cells with rCTGF and observed a decrease in LAMC2 expression to levels similar to those observed in the R182 WT cells (Figure 5D). To further validate the observed regulation of LAMC2 by CTGF, we analyzed the presence of secreted LAMC2 in the conditioned media from WT and CTGF KO cells. LAMC2 is a protein secreted during the process of ECM remodeling, and it is subjected to cleavage into a mature 100 KD form. As shown in Figure 5E, we observed a high expression of the processed LAMC2 (100 Kd) in the conditioned media from CTGF-KO cells, further supporting the role of CTGF in the regulation of ECM remodeling. 

To determine the functional significance of gaining LAMC2, we exposed WT R182 cells (anoikis sensitive) to conditioned media containing secreted LAMC2 collected from CTGF KO cells. As shown above, WT R182 cells are anoikis-sensitive, undergoing 75% cell death within 72 h of culturing in unattached conditions. The addition of conditioned media from CTGF KO cells to WT R182 cells was able to rescue anoikis, as demonstrated by a significant increase in viability at 24, 48, and 72 h (*p* ≤ 0.05, respectively) (Figure 5F). The addition of CTGF KO-conditioned media had no effect on the invasion capacity of WT cells. Taken together, these data demonstrate that CTGF negatively regulates LAMC2 expression and promotes sensitivity to anoikis.

### 3.5. Loss of CTGF Is Associated with Cytoskeleton Remodeling

**Figure 6:**
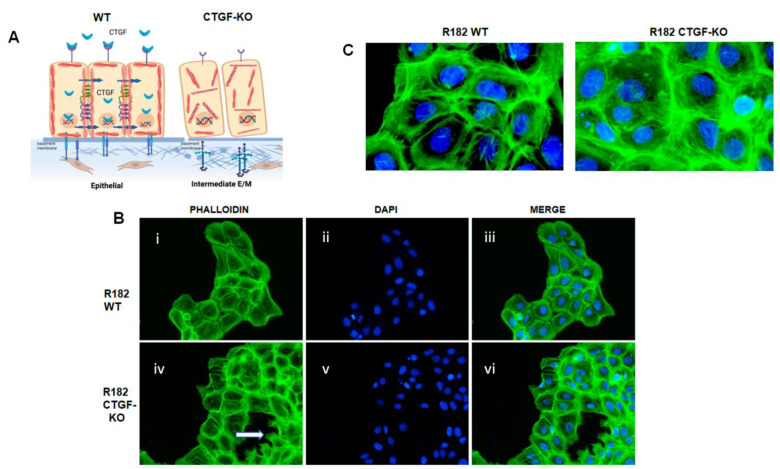
Loss of CTGF promotes extracellular matrix remodeling. (**A**) Proposed model of the role of CTGF in epithelial to mesenchymal cell transition in OC. (**B**) F-actin IF in R182 WT and R182 CTGF-KO cells demonstrates the presence of lamellopodia and (**C**) the reorganization of actin filaments. A representative figure of three independent experiments performed in triplicates.

Cytoskeleton remodeling is a critical step during the early EMT process, and is one of the main pathways that alters CTGF KO cells (Figure 6A). To determine whether CTGF expression is associated with cytoskeleton remodeling, we evaluated F-actin expression in R182 WT and CTGF-KO cells via IF. F-actin are the linear polymers of the G-actin subunit and make up the microfilaments in the cellular cytoskeleton [34]. Epithelial R182 cells show a uniform pattern of F-actin expression, mainly localized in the periphery and adjacent to the cell membrane (Figure 6(Bi–Biii),C). In R182-CTGF-KO cells, we observed higher intensity in F-actin staining, which is mostly cytoplasmic (Figure 6(Biv–Bvi),C). We also observed strong F-actin staining in the lamellipodia, present only in R182-CTGF-KO cells, which usually indicates cell migration (Figure 6(Biv)). These data further demonstrate a role for CTGF in maintaining an epithelial phenotype via the regulation of cytoskeleton remodeling, as shown in the model in Figure 6A. 

### 3.6. Loss of CTGF Alters Cell Stiffness

**Figure 7:**
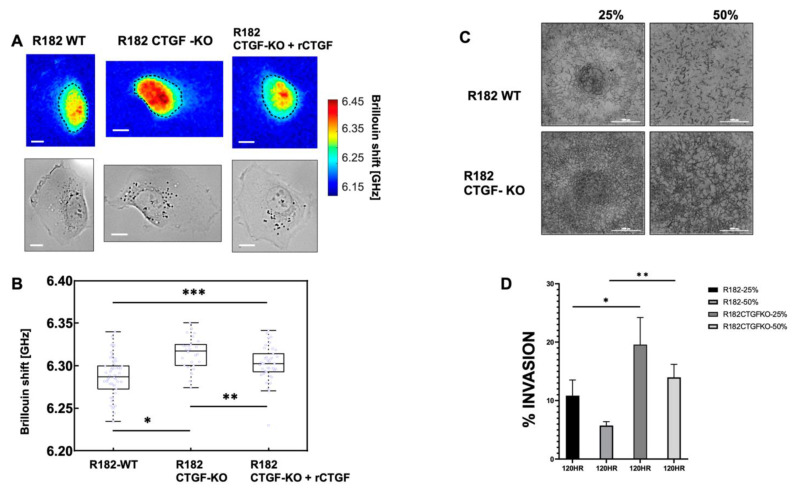
Cell stiffness of CTGF KO and WT R182 cells. (**A**) Top rows are Brillouin images. Bottom rows are co-registered bright-field images. Dashed line indicates the cell body. Scale bar: 10 µm. (**B**) Brillouin shift results. Multiple measurements were taken for each cell line; wt (n = 50); KO (n = 26); and rCTGF (n = 39). * *p* < 1.6 × 10^−5^. ** *p* = 0.015. *** *p* = 0.002. (**C**) Invasion assay performed with R182 WT and R182-CTGF KO with 25% and 50% Matrigel measured at 120 h. Independent experiments were performed in quadruplicate. Data are presented as mean ± SEM and an unpaired *t*-test was used to calculate statistical significance. Scale bar 1000 μm. (**D**) Representative images of invasion assay with R182 WT and CTGF KO cells in 25 and 50% Matrigel.

Cell stiffness is a mechanical property of a cell that is often related to adhesion, motility, differentiation, and invasiveness [35], and is measured using the gold standard of atomic force microscopy. More recently, optical Brillouin microscopy has been used to measure cell membrane stiffness [36,37]. This technique is an all-optical method and evaluates the mechanical properties of a cell utilizing Brillouin light scatter [37,38]. The Brillouin technology has been shown to be well correlated with the gold standard of atomic force microscopy in OC [39]. Cell stiffness is partly determined by the types of actin filaments and their organization. Because changes in the extracellular matrix may be associated with changes in cell stiffness, we measured the mechanical properties of R182 WT and R182 CTGF-KO cells utilizing Brillouin microscopy. The representative Brillouin images and the co-registered bright-field images of cells are shown in Figure 7A. We then used the averaged Brillouin shift of the cell body to represent the mechanical property of each cell. R182-CTGF KO cells demonstrated increased cell stiffness compared to R182 WT cells (*p* < 0.01). The addition of rCTGF (100 ng/mL) abrogated the increase in stiffness and returned the levels close to those of the WT cells (Figure 7B). We then determined the ability of R182 CTGF-KO cells to invade through matrix with increasing stiffness by culturing them in matrices with increasing concentrations of Matrigel (25, 50%). We found again that, at baseline, R182 CTGF- KO cells are more invasive than R182 WT cells and that they are able to maintain this invasive phenotype even with increasing stiffness of the matrix (Figure 7C,D). Taken together with the IF data demonstrating increased F-actin staining in CTGF-KO cells, the observed increase in cell stiffness suggests that cytoskeletal remodeling is an important outcome of CTGF loss in OC cells. 

### 3.7. Lack of CTFG Provides Tumorigenic Capacity

**Figure 8:**
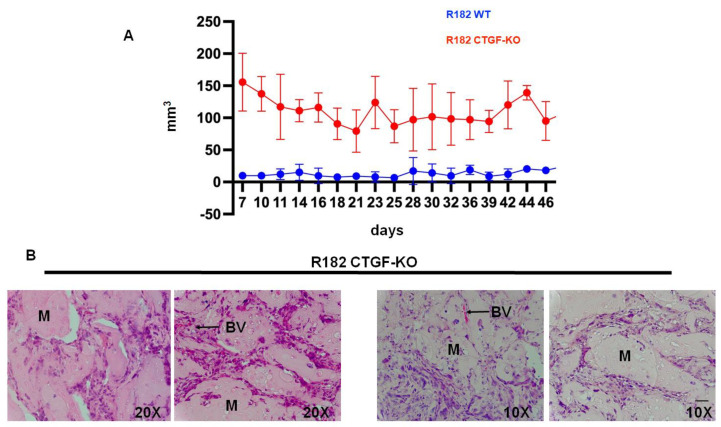
Tumorigenicity. (**A**) Tumor growth curves of R182 WT and R182 CTGF-KO cells in NCG mice. R182 CTGF-KO cells can form s.c. tumors while no detectable tumors were observed with R182 wt cells. (**B**) Histology of tumors formed by R182 CTGF-KO cells. Note cells invading the Matrigel (M) and the presence of neovascular process (BV). Representative figures of five independent animals. Scale bar: 100 μm.

Finally, we determined whether the characteristics observed in vitro following CTGF inhibition would provide cancer cells with a tumor formation capacity. Thus, 20 million R182 CTGF-KO or R182 WT OC cells were injected in the flank of NSG mice in 50% Hydrogel as described in the Materials and Methods section. Tumor growth was monitored with caliper measurement beginning 7 days after the injection of the cells. Mice were euthanized at 55 days post-injection and a histological analysis was performed to determine the presence of cancer cells. We observed consistently higher tumor volume in mice injected with R182 CTG-KO cells compared to mice injected with R182 WT cells which had no palpable tumor by the end of the study (Figure 8A). The histological analysis showed the presence of cancer cells within the Matrigel as well as the presence of new blood vessels in animals injected with R182 CTG-KO cells (Figure 8B). No tumors or presence of cancer cells were detected in the sites injected with R182 WT cells. 

## 4. Discussion

We describe, for the first time, the novel role of CTGF in maintaining an epithelial phenotype by regulating cytoskeleton remodeling and ECM interaction. While still maintaining their epithelial phenotype, cells lacking CTGF undergo reorganization of the cytoskeleton, reprogramming of the ECM, and acquisition of anoikis resistance, cell migration, and tumor formation capacity.

The epithelial phenotype is characterized by stable epithelial cell–cell junctions, apical–basal polarity, and interactions with the basement membrane [40]. These junctions are linked to the cytoskeleton, including a circumferential F-actin belt as well as keratins [41]. The interaction between epithelial cells and the ECM is essential for their apical–basal polarity and the maintenance of tissue homeostasis. The ECM is composed of a complex network of macromolecules that assemble into three-dimensional supramolecular structures that regulate cell growth, survival, motility, and differentiation by ligating specific receptors such as integrins, syndecans, and discoidin receptors [42]. An important aspect of the ECM is its high degree of plasticity and remodeling capacity specifically tailored to the structure/function and composition of each organ, and its configuration changes to reflect the physiological state of the tissue [12]. In this study, we found that one of the early events in the process of EMT is associated with ECM and cytoskeleton remodeling, two important cellular events controlling tumor formation [43].

Epithelial–mesenchymal transition (EMT) is a cellular process through which epithelial cells gain mesenchymal characteristics via the downregulation of their epithelial features [1]. For an epithelial cell to acquire a mesenchymal phenotype, it needs to be exposed to signals that originate from their microenvironment [15,43]. Luo [44] describes EMT as a result of the ecological adaptation to the external environment including the host immune system and stroma. As the epithelial cells undergo EMT, there are changes in gene expression and post-translational regulation mechanisms leading to the repression of these epithelial characteristics and the acquisition of mesenchymal characteristics [15]. EMT does not result in a single mesenchymal state, but rather results in multiple intermediate states with diverse levels of epithelial and mesenchymal features [16]. Recently, we described the characterization of the early stages of EMT and defined those cells as E/M hybrid state, which represents a stable state, with anoikis resistance and invasive capacity [15]. Using an in vitro model that recapitulates the acquisition of mesenchymal characteristics while maintaining the morphology and the expression of epithelial genes, we analyzed their transcriptome and identified CTGF as one of the genes that is differentially expressed between the epithelial and intermediate stage.

CTGF is a secreted matricellular protein with very complex biology, which modulates many signaling pathways primarily related to cell adhesion and migration, angiogenesis, myofibroblast activation, and extracellular matrix deposition and remodeling [45]. We found that the loss of CTGF in epithelial ovarian cancer cells is associated with modifications on the expression of several genes associated with the ECM, including LAMC2 SPP1, SV2A, RELN, COL6A3, and COL4A6. Changes in the ECM have been proposed to influence each of the classically defined and emerging hallmarks of cancer [43], including cell survival and metastasis. LAMC2 is a subunit of the heterotrimeric glycoprotein laminin-332, which is a fundamental component of epithelial basement membranes and regulates cell motility and adhesion [41]. High LAMC2 expression correlates with poorer survival in multiple cancers including thyroid, NSCLC, and cholangiocarcinoma [46,47]. Our data suggest that LAMC2 expression is directly regulated by CTGF, as shown by the significant increase in LAMC2 expression in cells lacking CTGF and the downregulation of LAMC2 expression following exposure to rCTGF. Interestingly, our data indicate a connection between CTGF-LAMC2 and anoikis resistance. CTGF-KO cells secrete high levels of LAMC2, which, if exposed to epithelial cells, will confer to them a resistance to anoikis. Indeed, LAMC expression has been reported to be a characteristic of a highly metastatic subpopulation of Tumor Initiating Cells (TIC) [48].

In ovarian cancer, molecular subgroups of HGSOC established by the TCGA identified a “mesenchymal” subgroup signature [49], which has been associated with the overexpression of EMT markers, the lowest rates of optimal surgical debulking, and worse OS [50,51]. LAMC2 expression was correlated with worse survival, lymph node metastasis, tumor-node-metastasis stages, and tumor status [46]. There are contradictory reports on the effect of CTGF in various malignancies [52,53,54,55,56]. Similar to our findings, Barbolina et al. [57] found the loss of CTGF to support OC invasion. Other investigators have described CTGF expression as a poor prognostic marker and promoting cancer cell invasion and metastasis [53,56,58,59]. One explanation for the diverse reports of CTGF activity is that the role of CTGF is dependent both on the tumor cell type as well as the tumor microenvironment. Our study focused on the role of CTGF in early EMT prior to the development of diffuse and advanced disease.

The results of our RNA seq data identifying extracellular matrix remodeling as the most significantly associated biological process with loss of CTGF suggest that one of the major mechanisms of CTGF function is its regulation of ECM remodeling. We postulate that the secretion of CTGF maintains the ECM necessary for the epithelial phenotype and the regulation of epithelial function. On the other hand, the loss of CTGF is associated with ECM remodeling towards a mesenchymal phenotype, as demonstrated by the expression of LAMC2 and reorganization of the actin filaments, both early steps in the EMT process. The mechanism by which CTGF regulates LAMC2 is currently unknown. One possible model is that the loss of CTGF releases the inhibition of the mesenchymal marker SNAIL/ZEB, promoting the remodeling of the extracellular matrix. Interestingly, no expression of TWIST was seen in the CTGF KO cells, suggesting that the loss of CTGF is an early step in EMT but alone does not support a fully mesenchymal phenotype. This hypothesis is also supported by the maintenance but not growth or metastasis of viable tumor cells in our in vivo experiments.

Finally, we show that the loss of CTGF increases the stiffness of epithelial R182 OC cells. Indeed, CTGF-KO cells are more invasive than WT cells even in a stiffer substrate. Consequently, it is plausible that the inhibition of CTGF expression leading to the remodeling of the ECM and the cytoskeleton modifies the stiffness of the cells, conferring migratory capacity. Given that cell stiffness measurements reflect the polymerization of F-actin, it is possible that not only the amount of F-actin, but the angle of the actin cytoskeleton and the stiffness of the ECM may be integral to the process of invasion.

The mechanisms regulating CTGF expression during this early process are still under investigation. CTGF is a validated downstream target of YAP, a key regulator of cell surface and substrate stimuli including cell stiffness. Although we did not see an increase in YAP expression in CTGF KO cells, one possible hypothesis is that the loss of CTGF increases the nuclear localization of YAP, thus increasing cell stiffness. Experiments are currently ongoing to test this hypothesis. 

## 5. Conclusions

In conclusion, we demonstrate that the loss of CTGF is one of the early events leading to EMT. We demonstrate that, following the inhibition of CTGF expression, epithelial cells undergo a process of cytoskeleton remodeling and expression of a different type of ECM, one that will promote their differentiation into an intermediate stage, maintaining some of the epithelial phenotype but acquiring mesenchymal characteristic such as anoikis-resistant tumor formation and migration capacity. Further studies evaluating the upstream regulators and downstream targets of CTGF may provide novel therapeutic targets that can curtail the metastatic process in ovarian cancer.

## Data Availability

Once the manuscript is accepted for publication, all the data obtained from the RNA seq will be deposited and made available.

## Supplementary Materials

The following supporting information can be downloaded at: https://www.mdpi.com/article/10.3390/cancers15194834/s1, File S1: Full blots for figures; Table S1: CTGF KO vs WT pathways and GO terms.
