# Supplementary material for: A Novel Role of Connective Tissue Growth Factor in the Regulation of the Epithelial Phenotype
Source: Cancers (Basel). 2023 Oct 2;15(19):4834. doi: 10.3390/cancers15194834 (PMC10571845; doi:10.3390/cancers15194834)

2A: anti-CTGF

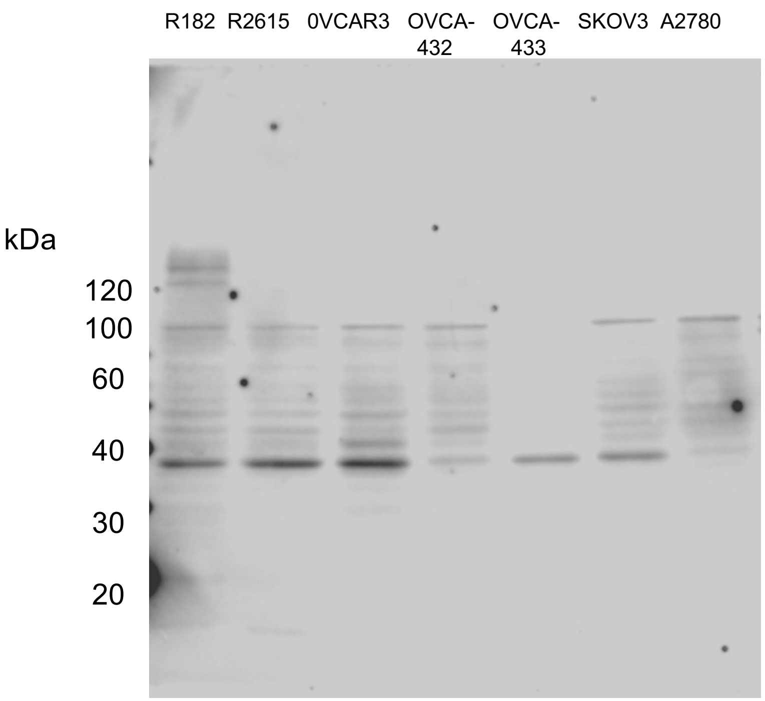

2A: anti-GAPDH

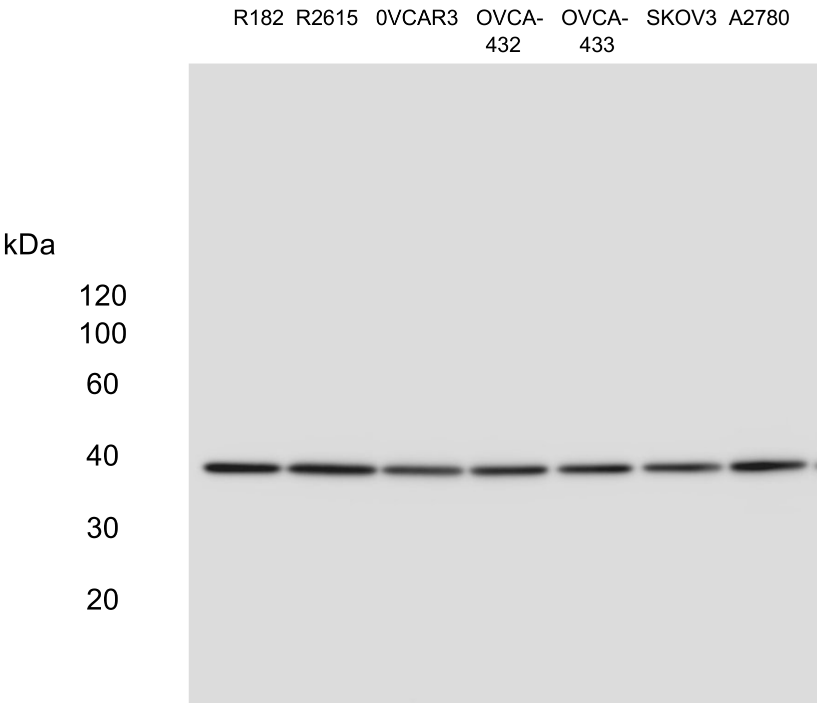

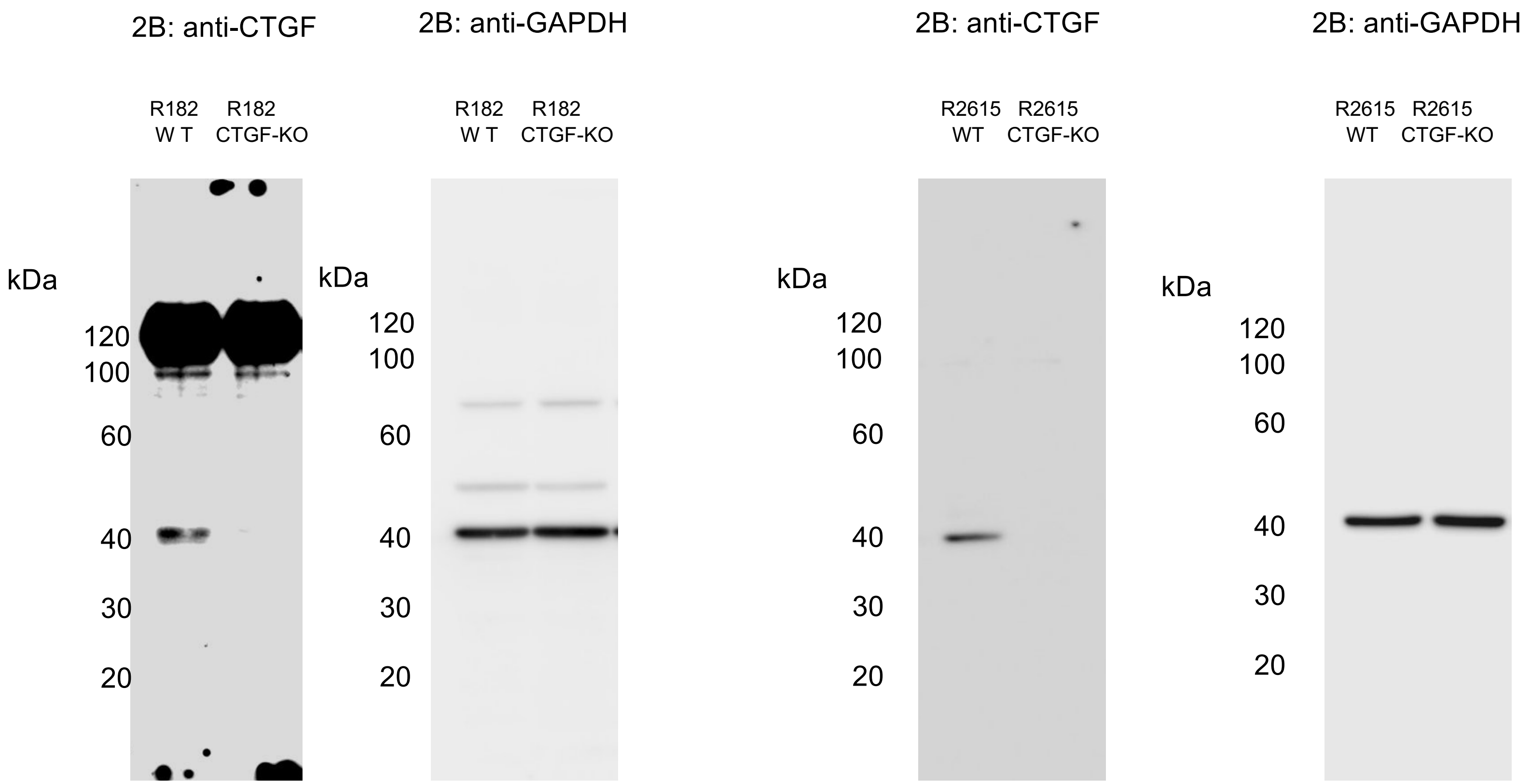
2B: anti-CTGF
R182 W T
R182 CTGF-KO
kDa
120
100
60
40
30
20
2B: anti-GAPDH
R182 W T
R182 CTGF-KO
kDa
120
100
60
40
30
20
2B: anti-CTGF
R2615 WT
R2615 CTGF-KO
kDa
120
100
60
40
30
20
2B: anti-GAPDH
R2615 WT
R2615 CTGF-KO
kDa
120
100
60
40
30
20

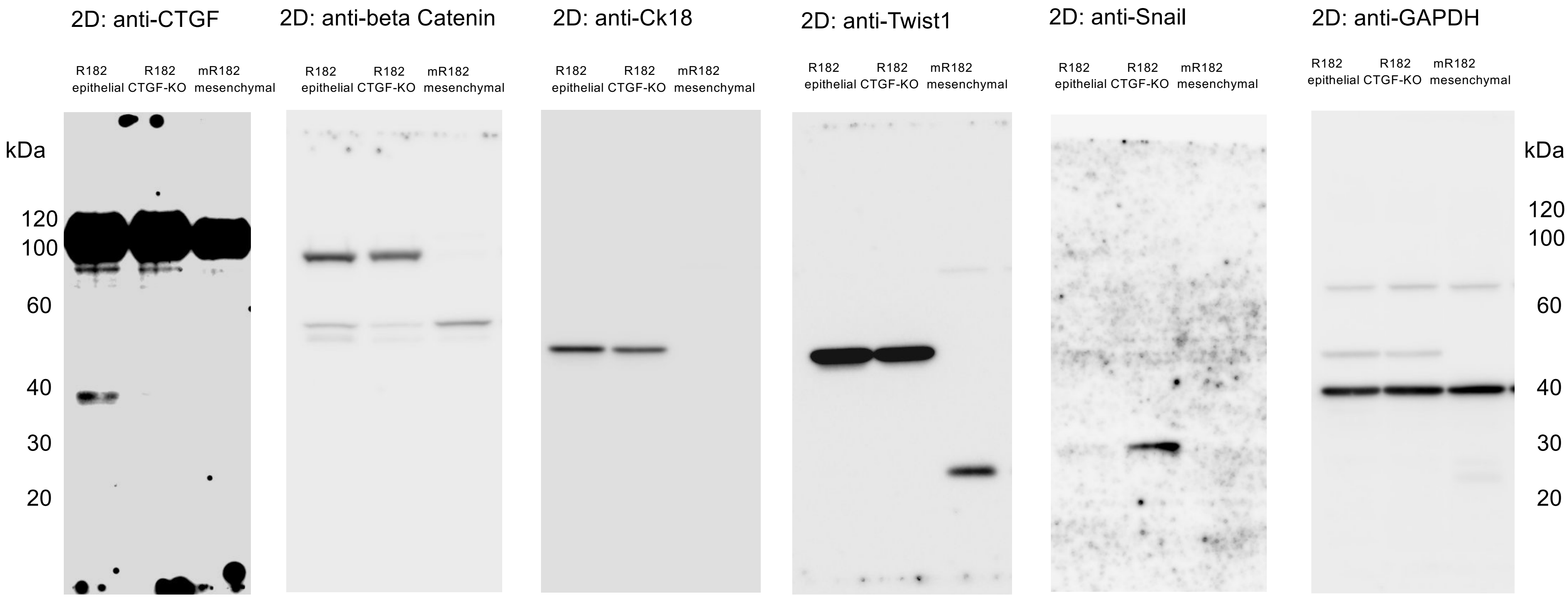

2D: anti-CTGF
R182 epithelial
R182 CTGF-KO
mR182 mesenchymal
kDa
120
100
60
40
30
20
2D: anti-beta Catenin
R182 epithelial
R182 CTGF-KO
mR182 mesenchymal
2D: anti-Ck18
R182 epithelial
R182 CTGF-KO
mR182 mesenchymal
2D: anti-Twist1
R182 epithelial
R182 CTGF-KO
mR182 mesenchymal
2D: anti-Snail
R182 epithelial
R182 CTGF-KO
mR182 mesenchymal
2D: anti-GAPDH
R182 epithelial
R182 CTGF-KO
mR182 mesenchymal
kDa
120
100
60
40
30
20

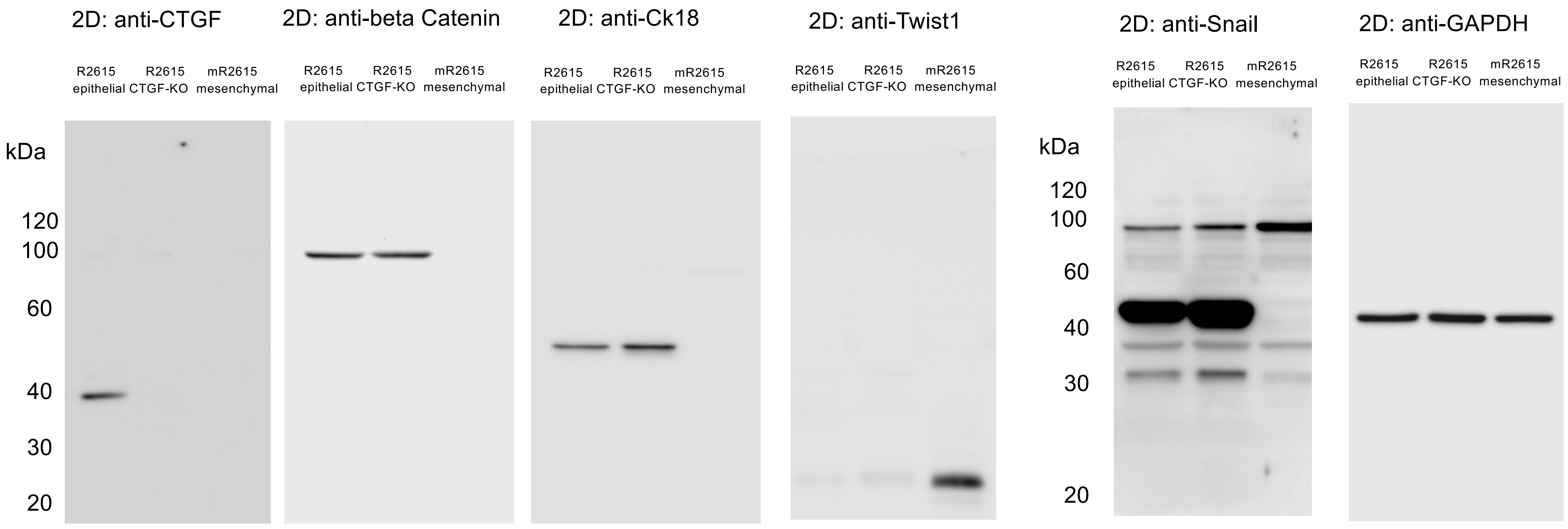
2D: anti-CTGF
2D: anti-beta Catenin
2D: anti-Ck18
2D: anti-Twist1
2D: anti-Snail
2D: anti-GAPDH
R2615 epithelial
R2615 CTGF-KO
mR2615 mesenchymal
kDa
120
100
60
40
30
20

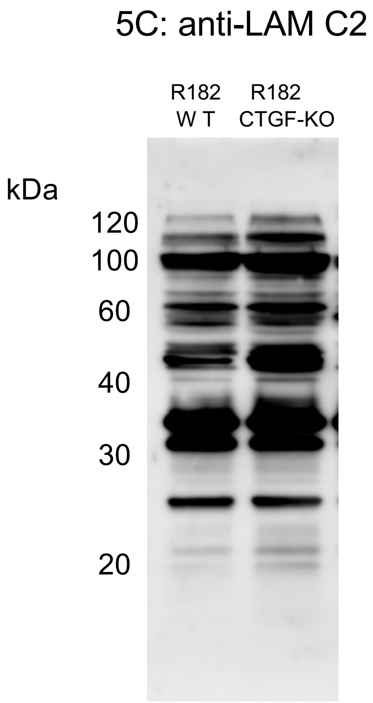
5C: anti-LAM C2
R182 W T
R182 CTGF-KO
kDa
120
100
60
40
30
20

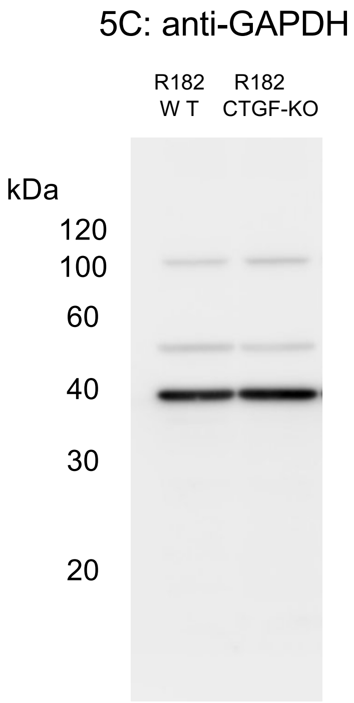
5C: anti-GAPDH
R182 W T
R182 CTGF-KO
kDa
120
100
60
40
30
20

5E: anti-LAM C2

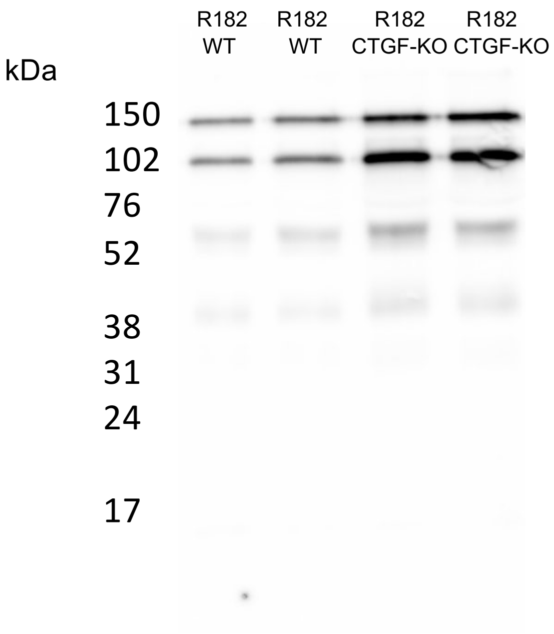

Supplement: Supplementary file 1 [file cancers-15-04834-s001.zip › Supplementary File S1. Full blots for figures.pdf]
